# Medium/Long wavelength sensitive opsin diversity in Pitheciidae

**DOI:** 10.1038/s41598-017-08143-2

**Published:** 2017-08-10

**Authors:** Vinicius D. L. R. Goulart, Jean P. Boubli, Robert J. Young

**Affiliations:** 1 CAPES Foundation, Ministry of Education of Brazil, Brasília, DF 70040-020 Brazil; 2 School of Environment and Life Sciences, Peel Building, University of Salford Manchester, Salford, M5 4WT UK

## Abstract

New World primates feature a complex colour vision system. Most species have polymorphic colour vision where males have a dichromatic colour perception and females can be either dichromatic or trichromatic. The adaptive value of high allelic diversity of opsins, a light sensitive protein, found in primates’ eyes remains unknown. Studies revealing the allelic diversity are important as they shed light on our understanding of the adaptive value of differences in the colouration of species and their ecologies. Here we investigate the allelic types found in Pitheciidae, an understudied New World primate family, revealing the diversity of medium/long wavelength sensitive opsins both in cryptic and conspicuous species of this primate family. We found five alleles in *Cacajao*, six in Callicebinae (i.e. *Plecturocebus, Cheracebus*, and *Callicebus*), four in *Chiropotes*, and three in *Pithecia*, some of them reported for the first time. Both cryptic and conspicuous species in this group presented high allelic diversity.

## Introduction

While most terrestrial mammals possess dichromatic colour perception, similar to a red-green colour blind human, primates may exhibit: (1) monochromatic colour vision, which occurs in several nocturnal species; (2) routine dichromatic colour vision in tarsiers and some lemur species; (3) routine trichromatic colour vision, mostly in Old World primates; and (4) polymorphic colour vision in most New World primates where males are obligatory dichromats, whereas females can have either dichromatic or trichromatic colour vision systems^1–5^. Strepsirrhine primate species have a potential polymorphic colour vision, however, this primate suborder lacks behavioural studies confirming a polymorphic colour vision as possessed by New World primates^6, 7^. This variation in colour vision in New World primates arises from a single locus for the gene coding the medium-long (M/L) wavelength sensitive opsin on the X chromosome^8, 9^; that is, males are obligatory dichromats as they are hemizygotes, and females can be either dichromats (i.e. homozygotes) or trichromats (i.e. heterozygotes)^10^. In New World primates, allelic diversity of the Medium-Long wavelength sensitive opsin results in intraspecific variation in colour vision perception^11^.

The functional role of opsin polymorphism is still not completely understood; however, both trichromatic and dichromatic colour vision phenotypes are proven to have different behavioural advantages. Dichromatic individuals are better at detecting camouflaged stimuli and seeing in low light levels, whereas ripe food and new leaves are better detected by trichromats^12–16^. Social signals, observed on bare skin, such as emotional states, sexual signals, threat displays are cited as factors leading to the evolution of routine trichromatic colour vision^17^. Undoubtedly, bare skin is a relevant source of information, which might not be perceived by individuals with dichromatic colour vision. Even species that possess trichromatic colour vision might have difficulty to differentiate colours if the M/L opsin alleles in a heterozygotic female are separated by less than 10 nm^18^. Anomalous trichromacy (i.e., impaired colour vision but not complete loss) is frequent in howler monkeys and might be common in highly polymorphic colour vision species^19^.

Uakaries, genus *Cacajao*, are Neotropical primate species occurring in the upper Amazonian region and they are represented by two contrasting colour phenotypes, one red-faced (i.e. *Cacajao calvus*) and the other black-faced (*Cacajao ayresi, Cacajao hosomi, Cacajao melanocephalus*)^20^. Uakaris are seed-eating specialist species living in groups with up to 200 individuals in a fission-fusion society^21–25^. *Cacajao* was, until recently, a major lineage not investigated regarding its colour vision. It is now known that *Cacajao calvus* has high allelic diversity of opsins^26^. The bare face of the bald red-uakari is thought to be related to communication among group members. Its simple one coloured face could allow for efficient communication through facial movements and expressions^27^. Importantly, the red colourful display on the bald uakari’s face may present variations in haemoglobin pigmentation, which might indicate emotional states or health status^17, 28^. In addition, the bald uakari’s vision was recently reported as being highly polymorphic with a six functional alleles for the medium-long wavelength sensitive opsin. This makes the species an interesting subject to test the importance of reddish displays on the evolution of colour vision. Interestingly, the cryptic black-faced congeneric (i.e. *Cacajao melanocephalus*, *Cacajao ayresi* and *Cacajao hosomi*) lack a bare face. To date, nothing is known about the colour vision in this species and this is one of the objectives of the present study.

The genus *Chiropotes*, also possesses species with red skin exposed on the face (*Chiropotes albinasus*), whereas other species in the genus do not possess this characteristic^29^. These medium sized primates are secretive and difficult to observe in the wild; this is why there are few studies on their behaviour^30^. However, it is known that *Chiropotes* form fission-fusion groups with highly affiliative behaviour, and males are more gregarious and tolerant towards juveniles than females^30, 31^. One *Chiropotes* species (*Chiropotes utahickae*) was investigated with regards to its colour vision and three M/L opsin alleles were found, also possessing a colour vision similar to other New World primates^32^.

*Pithecia* is a cryptic species and lives in small group sizes (i.e. 4 to 6 individuals)*;* however, their diet is similar to *Cacajao* and *Chiropotes* ^33^. In the Pitheciidae, the genus *Pithecia* presents the highest degree of sexual dichromatism^30^. This is a relevant factor when studying the polymorphic colour vision system in New World primates, especially considering the importance of this characteristic in relation to visual communication. This genus also possesses a polymorphic colour vision system as observed in other New World primates with three different known alleles for the M/L opsin gene^32^.

In Callicebinae, the genus *Plecturocebus* was also found to possess a relatively high number of alleles for M/L opsins. While most New World primates have three types of photopigments for the medium or long wavelength, *Plecturocebus moloch* have five cone types in a range from 530 to 562 nm^11^. Despite similarities in the higher allelic diversity, *Cacajao* and *Plecturocebus* are contrasting in many ways. Callicebinae is the New World primate subfamily with the greatest number of species (33 recognised species)^34, 35^. Despite the number of species, it is one of the least studied primate subfamilies^36^. Titi monkeys are small (1.5 kg) with timid behaviour spending most of the time under umbrella-like canopy, forming family groups commonly of five individuals with a diet consisting of leaves and fruits^36–38^. Some species show countershading coloration with a bright coppery colour. It is assumed that this colouration is not visible to dichromatic colour vision individuals and the role of this conspicuous colouration is still unknown^39^. Interestingly, only two species have been investigated so far regarding their colour vision: *Plecturocebus brunneus* and *Plecturocebus moloch*.

There are three main methods employed to determine colour vision perception; behavioural studies, direct physiological measurements and molecular analysis of opsin genes^40, 41^. In the behavioural approach, animals are trained to select colour referenced stimuli in discrimination experiments, which evaluate the degree of difficulty to detect the colour stimuli^41^. In the physiological approach, an electroretinogram where the spectral sensitivity of photoreceptors in the retina is measured or spectrophotometry of *in vitro* reconstituted photopigments from cDNA are measured^8^. Molecular analysis is the most widely employed method. From molecular analysis, it is possible to infer the peak of sensitivity of the expressed opsin gene by verifying amino acid changes at specific sites^42^. The combination of both molecular and ecological data promises to provide new insights on the role of colour vision evolution in primates^43^.

The maintenance of such a high number of alleles in this family, strongly, suggests that it has an adaptive function. Here we evaluate, qualitatively, the allelic diversity in the family Pitheciidae (*Cacajao*, *Plecturocebus, Cheracebus, Callicebus, Chiropotes*, and *Pithecia*).

## Results

All individuals had their sex confirmed by molecular analysis. By analysing the sites 180 at the exon 3 and sites 277, 285, and 294 at the exon 5 we were able to find six allelic variants of the M/L opsin gene (Tables 1 and 2). We found five alleles in the *Cacajao* genus (532, 545, 550, 555, 560 λ_max_nm). We found *C. ayresi* with the alleles AFA (532 λ_max_nm), AYT (555 λ_max_nm) and SYT (560 λ_max_nm); *C. hosomi* with the alleles AFA, AFT (545 λ_max_nm) and SYT; *C. melanocephalus* with AFT, AYT and SYT. Thus, in total, they had a total of five different alleles for the M/L opsin gene. These three *Cacajao* species have highly pigmented skin. *C. calvus*, which has exposed red facial skin, had four of these alleles, namely, AFA, AFT, SFT and SYT.

**Table 1:** Observed numbers of alleles of Medium/Long wavelength sensitive opsin in Pitheciidae primate species.

Species	Allele	SYT	AYT	SFT	AFT	SFA	AFA
Expected λ_max_ nm		560	555	550	545	534	532
*Cacajao*	*ayresi*	1	1				1
	*calvus*	3		1	1		2
	*hosomi*	1			2		1
	*melanocephalus*	1	3		1		
*Plecturocebus*	*cinerascens*	4	3	1			
	*bernhardi*	1	2		1		
	*miltoni*			1	3		
	*moloch*			1	3		
*Cheracebus*	*lugens*	1	3	1	2	1	4
*Callicebus*	*nigrifrons*	4	6	1	1		
*Chiropotes*	*albinasus*	1					
	*israelita*		1		2		2
	*satanas*						1
*Pithecia*	*irrorata*		1		1		1
*Pithecia*	*pithecia*				1

**Table 2:** List of species and medium/long wavelength sensitive genotypes found in Pitheciidae.

Species	Sex	180	277	285	294	Allele		Provenance
*Cacajao ayresi*	Female	A	F	A	N	AFA		Araçá River, Amazonas, Brazil
*Cacajao ayresi*	Female	A/S	Y	T	N	AYT	SYT	Araçá River, Amazonas, Brazil
*Cacajao calvus*	Male	S	Y	T	N	SYT		Sacado do Tarauacá, Acre, Brazil
*Cacajao calvus*	Male	A	F	A	N	AFA		Jutai River, Amazonas, Brazil
*Cacajao calvus*	Male	A	F	A	N	AFA		Jutai River, Amazonas, Brazil
*Cacajao calvus*	Female	A/S	F	T	N	AFT	SFT	Jutai River, Amazonas, Brazil
*Cacajao calvus*	Female	S	Y	T	N	SYT		Jutai River, Amazonas, Brazil
*Cacajao calvus*	Male	S	Y	T	N	SYT		Jutai River, Amazonas, Brazil
*Cacajao hosomi*	Male	S	Y	T	N	SYT		Serra do Imeri, Xamata, Amazonas, Brazil
*Cacajao hosomi*	Female	A	F	T/A	N	AFT	AFA	Serra do Imeri, Xamata, Amazonas, Brazil
*Cacajao hosomi*	Male	A	F	T	N	AFT		Salto Hua, Amazonas, Brazil
*Cacajao melanocephalus*	Male	A	Y	T	N	AYT		Amanã Lake, Amazonas, Brazil
*Cacajao melanocephalus*	Female	A	Y/F	T	N	AYT	AFT	Manacapuru, Amazonas, Brazil
*Cacajao melanocephalus*	Female	A/S	Y	T	N	AYT	SYT	Santa Isabel do Rio Negro, Amazonas, Brazil
*Plecturocebus cinerascens*	Female	S	Y	T	N	AYT		Aripuana, Amazonas, Brazil
*Plecturocebus cinerascens*	Male	S	F	T	N	SFT		Pimenta Bueno, Rondônia, Brazil
*Plecturocebus cinerascens*	Female	A/S	Y	T	N	AYT	SYT	Pimenta Bueno, Rondônia, Brazil
*Plecturocebus cinerascens*	Male	S	Y	T	N	SYT		Pimenta Bueno, Rondônia, Brazil
*Plecturocebus cinerascens*	Female	S	Y	T	N	SYT		Pimenta Bueno, Rondônia, Brazil
*Plecturocebus cinerascens*	Female	A/S	Y	T	N	AYT	SYT	Cabixi, Rondônia, Brazil
*Plecturocebus bernhardi*	Male	A	Y	T	N	AYT		Aripuana, Amazonas, Brazil
*Plecturocebus bernhardi*	Female	A	F	T	N	AFT		Aripuana, Amazonas, Brazil
*Plecturocebus bernhardi*	Male	A	Y	T	N	AYT		Aripuana, Amazonas, Brazil
*Plecturocebus bernhardi*	Female	S	Y	T	N	SYT		Machadinho D’Oeste, Rondônia, Brazil
*Cheracebus lugens*	Female	A	Y	T	N	AYT		São Gabriel da Cachoeira, Amazonas, Brazil
*Cheracebus lugens*	Female	A	F	T/A	N	AFT	AFA	Ig Mandiquie, Amazonas, Brazil
*Cheracebus lugens*	Female	S	F	T/A	N	SFT	SFA	Rio Marauia, Amazonas, Brazil
*Cheracebus lugens*	Female	A	Y	T	N	AYT		Balawau, Amazonas, Brazil
*Cheracebus lugens*	Female	A	F	A	N	AFA		Marari, Amazonas, Brazil
*Cheracebus lugens*	Female	A	F	A	N	AFA		Ig Anta, Amazonas, Brazil
*Cheracebus lugens*	Male	A	Y	T	N	AYT		Ig Anta, Amazonas, Brazil
*Cheracebus lugens*	Male	A	F	A	N	AFA		Ig Cuieiras, Amazonas, Brazil
*Cheracebus lugens*	Female	S	Y/F	T/A	N	*		São Gabriel da Cachoeira, Amazonas, Brazil
*Cheracebus lugens*	Female	S	Y	T	N	SYT		São Gabriel da Cachoeira, Amazonas, Brazil
*Cheracebus lugens*	Male	A	F	T	N	AFT		Marari, Amazonas, Brazil
*Plecturocebus miltoni*	Male	A	F	T	N	AFT		Aripuana, Amazonas, Brazil
*Plecturocebus miltoni*	Female	A/S	F	T	N	AFT	SFT	Aripuana, Amazonas, Brazil
*Plecturocebus miltoni*	Male	A	F	T	N	AFT		Aripuana, Amazonas, Brazil
*Plecturocebus moloch*	Female	A/S	F	T	N	AFT	SFT	Alta Floresta, Mato Grosso, Brazil
*Plecturocebus moloch*	Male	A	F	T	N	AFT		Alta Floresta, Mato Grosso, Brazil
*Plecturocebus moloch*	Male	A	F	T	N	AFT		Alta Floresta, Mato Grosso, Brazil
*Callicebus nigrifrons*	Male	A	Y	T	N	AYT		Minas Gerais, Brazil
*Callicebus nigrifrons*	Female	A/S	Y	T	N	AYT	SYT	Minas Gerais, Brazil
*Callicebus nigrifrons*	Male	A	F	T	N	AFT		Minas Gerais, Brazil
*Callicebus nigrifrons*	Female	A	Y	T	N	AYT		Minas Gerais, Brazil
*Callicebus nigrifrons*	Male	S	F	T	N	SFT		Minas Gerais, Brazil
*Callicebus nigrifrons*	Female	A/S	Y	T	N	AYT	SYT	Minas Gerais, Brazil
*Callicebus nigrifrons*	Female	A/S	Y	T	N	AYT	SYT	Minas Gerais, Brazil
*Callicebus nigrifrons*	Female	A	Y	T	N	AYT	SYT	Minas Gerais, Brazil
*Chiropotes albinasus*	Female	S	Y	T	N	SYT		Maués Açú River, Amazonas, Brazil
*Chiropotes israelita*	Female	A	Y/F	T	N	AYT	AFT	Ig Anta, Amazonas, Brazil
*Chiropotes israelita*	Female	A	F	A	N	AFA		Ig Anta, Amazonas, Brazil
*Chiropotes israelita*	Female	A	F	T	N	AFT		Marauia River, Amazonas, Brazil
*Chiropotes israelita*	Male	A	F	A	N	AFA		Demeni River, Amazonas, Brazil
*Chiropotes satanas*	Male	A	F	A	N	AFA		Unknown locality
*Pithecia irrorata*	Female	A	F	A	N	AFA		Autazes, Amazonas, Brazil
*Pithecia irrorata*	Female	A	Y/F	T	N	AYT	AFT	Jari Lake, Amazonas, Brazil
*Pithecia pithecia*	Male	A	F	T	N	AFT		Manaus, Amazonas, Brazil

We found 6 alleles in Callicebinae (532, 534, 545, 550, 555, 560 λ_max_nm)*. P. bernhardi* with AFT, AYT, and SYT; *C. cinerascens* with SYT, AYT, and SFT (550 λ_max_nm); *C. lugens* with AFA, SFA, AFT, SFT, AYT and SYT; *P. miltoni* with SFT and AFT; *P. moloch* with AFT and SFT; and *C. nigrifrons* with SFT, AYT, AFT, and SYT. It was not possible to identify the allele in one individual of *C. lugens* due to double heterozygous sites at 277 and 285.

We found four alleles in *Chiropotes* (532, 545, 555, 560 λ_max_nm). *C. albinasus* with allele SYT; *C. israelita* with the alleles AFA, AFT, AYT and SYT; and *C. satanas* with allele AFA. In *Pithecia*, three alleles (532, 545, 555 λ_max_nm): *P. irrorata* with AFA, AFT and AYT and *P. pithecia* with AFT.

All sequences were identified from repeated sequencings from both strands and independent PCR reactions. Sequences are available in GenBank (Accession numbers KY345056-KY345113).

## Discussion

The results reported here show that the diversity of M/L opsins found in *Cacajao, Callicebus, Plecturocebus, Cheracebus, Chiropotes*, and *Pithecia* is greater than previously reported. Although the samples’ origins and the sample sizes were not appropriate to accurately estimate the opsin frequency, our results contribute to the knowledge of colour vision polymorphism in New World primates. Some allelic variants were found in only one individual per species requiring further studies for confirmation. For instance, the allele SFA was found in one specimen of *Cheracebus lugens*, and we suggest further confirmation.

We found three alleles in *C. ayresi*, three in *C. hosomi*, and three in *C. melanocephalus*. The different alleles found in this species group indicates that the black-headed uakaris have similar high allelic diversity as found in the polymorphic red-faced uakari^26^. Further studies of allelic frequencies with black-headed morphs might confirm a highly polymorphic colour vision, suggesting that the red colourful display found in *C. calvus* is not the ultimate cause for the high number of allelic variants of the Medium/Long wavelength sensitive opsin in New World primates. Thus, challenging the importance of socio-sexual displays in the evolution of routine trichromatic colour vision^17^. To the best of our knowledge, this is the first report on the opsin diversity of black-headed uakaris (i.e. *Cacajao ayresi, Cacajao hosomi, Cacajao melanocephalus*).

From physiological studies using electroretinogram flicker photometry, *Plecturocebus* was reported to have the highest number of opsin alleles within a species (*P. moloch*), showing five different M/L photopigments in captive individuals^11^. In a study with wild populations of *P. brunneus* using a molecular approach, three alleles were found representing the most common types from the aforementioned research (i.e. AFA, AFT, and SYT)^44^. These three alleles were found for the Callicebinae subfamily in our analysis. In addition, from the two individuals of *P. moloch* investigated here, the allelic variant SFT was found representing an absorbance peak of 550 λ_max_nm. Considering the subfamily Callicebinae only, we have evidence of six different alleles, which increases the known opsin diversity found.

In the genus *Chiropotes*, in addition to the alleles AFA, AFT, and SYT reported in the literature^32^, we found an additional variant AYT with a peak spectral sensitivity of 555 λ_max_nm in *Chiropotes israelita*, increasing the number of functional alleles from three to four. Furthermore, the genus *Pithecia* was reported with the alleles AFA, AFT, and SYT^45^. We found a variant AYT allele with the sensitivity peak of 555 λ_max_nm, increasing the number of known opsin alleles from three to four.

Spectral shifts in opsin sensitivity could also result from mutations in other sites of the M/L opsin gene^46^. However, the M/L opsin sensitivity peaks are best explained by the “three-site-rule”^47^. Now that this variation in Pitheciidae is known, one possible approach is to confirm the sensitivity of this protein *in vitro*. cDNA can be used to produce functioning opsins *in vitro* by cloning in cultured cells to measure the photopigment sensitivity by spectrophotometric measurements^48^. Alternatively, electroretinography from Pitheciidae species in captivity would help to confirm the high allelic variation found.

Similarly to Corso *et al*.^26^, we found no evidence of routine trichromatic colour vision in the red-faced uakari. Additionally, we also found no evidence of routine trichromatic colour vision in the black-headed uakaris. If routine trichromatic colour vision was found in the red-faced uakari, as in *Alouatta* ^49^, this would support the importance of socio-sexual signals in the evolution of colour vision in primates^17^, which was not the case. However, both cryptic and conspicuous *Cacajao* morphs share high opsin diversity. This results in an increased number of heterozygotes and potentially more trichromatic females in a group. Similarly in Callicebinae (a subfamily in which most of its skin is covered with fur) shows high allelic diversity again resulting in a high proportion of trichromatic females.

Despite the fact that trichromatic colour vision is best suited to distinguish colour modulations on the skin^17^, there is evidence that the ability to discriminate red colours in primate vision evolved after red visual traits in primate species^50^. For example, primate species that are able to discriminate red-green hues have less red fur than dichromate species^51^. Other species, such as *Chiropotes albinasus*, have strong red facial marking, but do not have routine trichromatic colour vision. Geographical and ecological factors may also affect the morphology of primate species. Group size and incidence of UV light may lead to more complex faces and dark facial masks^27^. Thus, variations in facial colouration are expected to be generated from both social and biogeographical pressures.

The exaggerated reddish displays in uakaris and the coppery coloration in Callicebinae could be an evolutionary adaptation to allow dichromatic colour vision individuals to identify social signals. Further studies measuring skin and fur colouration would be useful to understand the role of exaggerated reddish displays in New World primates, for instance, if variants in the red-faced uakari are detectable by dichromatic colour vision individuals. This would indicate why these species show this exaggerated colouration pattern. Future research should focus on the benefits of primate groups possessing both dichromatic and trichromatic individuals rather than focusing only on the consequences of different colour vision on an individual primate.

## Methods

### DNA extraction

Genomic DNA was extracted from muscle tissue, deposited in museum collections (Table 3), using DNeasy blood and tissue kit (QIAGEN) following the manufacture’s protocol without modifications. The extracted DNA was quantified using a NanoDrop 2000 (Thermo Scientific) to rule out allelic dropouts (i.e. sequencing one chromosome only in heterozygous individuals) by using samples with a DNA concentration higher than 200 picograms/microliter^52, 53^. We used 1 μL of extracted DNA in buffer AE to obtain 260 nm readings providing the DNA concentration, which were, on average 20000 pg/μl.

**Table 3:** List of species used in the molecular analysis of allelic types of Medium/Long visual photopigments.

Species	Female	Male	Total
*Cacajao ayresi*	2	0	2
*Cacajao calvus*	2	4	6
*Cacajao hosomi*	1	2	3
*Cacajao melanocephalus*	2	1	3
*Plecturocebus cinerascens*	4	2	6
*Plecturocebus bernhardi*	2	2	4
*Cheracebus lugens*	8	3	11
*Plecturocebus miltoni*	1	2	3
*Plecturocebus moloch*	1	1	2
*Callicebus nigrifrons*	5	3	8
*Chiropotes albinasus*	1	0	1
*Chiropotes israelita*	3	1	4
*Chiropotes satanas*	0	1	1
*Pithecia irrorata*	2	0	2
*Pithecia pithecia*	0	1	1
Total	34	23	58

### Sex Assignment

To confirm the primates’ sex from all samples, Polymerase Chain Reactions (PCR) were conducted using the primers for the Amelogenin in the X chromosome (Forward: 5′–ACCACCAGCTTCCCAGTTTA–3′; and Reverse: 5′–GCTGGGWTAGAACCAAGCTG–3′) for a _~_200 bp fragment, and the Y-linked sex-determining region (SRY) (Forward: 5′–AGTGAAGCGACCCATGAACG–3′; and Reverse: 5′–TGTGCCTCCTGGAAGAATGG–3′) for a _~_165 bp fragment^54^. A 25 μL PCR was performed 0.25 μL of TaKaRa Ex Taq® Hot Start Version (1.25 units), with 2.5 μL of × 10 Ex Taq buffer, 4 μL of 2.5 mM dNTP mixture, 0.8 μL of each primer at 100 pmol/μL, 3 μL of template DNA, and 14 μL of pure PCR water to complete the final volume. All reactions were performed with a negative control using pure PCR water instead of DNA template.

The thermal cycling profile followed one cycle of initial denaturation at 94 °C for two minutes; then 40 cycles of denaturation at 94 °C for 30 seconds, annealing at 58 °C for 30 seconds, and elongation at 72 °C for 30 seconds; lastly, a final elongation cycle of 72 °C for 5 minutes. Amplifications were confirmed by electrophoresis in a 1.3% agarose gel, using HyperLadder I™ (Bioline) as reference. The amplification of the Amelogenin, gene present in all samples at the X chromosome, was used as a positive control for all reactions and confirmed through molecular weight. The presence of two bands (i.e. one for the X and one for the Y chromosome) in the agarose gel allowed the assignment of males, while one band determinate females (i.e. one band for the X chromosome) through a benchtop verification^54^.

### Amplification and sequencing

Primers for the Exon 3 were: Forward 5′–CTCCAACCAAAGATGGGCGG–3′; Reverse 5′–ATCACAGGTCTCTGGTCTCTG–3′. Primers for the Exon 5 were: Forward 5′–GAATCCACCCAGAAGGCAGAG–3′; Reverse 5′–ACGGGGTTGTAGATAGTGGCA–3′. PCRs were carried out using 0.25 μL of TaKaRa Ex Taq® Hot Start Version (1.25 units), with 2.5 μL of × 10 Ex Taq buffer, 4 μL of 2.5 mM dNTP mixture, 0.25 μL of each primer at 100 pmol/μL, 0.5 μL of BSA (New England BioLabs) 3 μL of template DNA, and 14.25 μL of pure PCR water to a final 25 μL reaction. Negative controls were employed in all reactions using pure PCR water instead of DNA template. The Exon 3 thermocycling profile consisted of one initial cycle of 98 °C for five minutes; 40 cycles of 98 °C for 30 seconds, 62 °C for 30 seconds decreasing 0.1 °C per cycle, and 72 °C for 30 seconds; followed by a final elongation cycle of 72 °C for 5 minutes. The Exon 5 used the same thermocycling profile, but the annealing temperature was 60 °C decreasing 0.1 °C per cycle. PCR clean-up and sequencing were performed independently by Source Bioscience Sequencing commercial service (Rochdale, UK) using Applied Biosystems 3730 series DNA Analysers.

### Genotype determination

Amino acid changes at site 180 in Exon 3, 277 and 285 in Exon 5 are responsible for major shifts in the peak absorbance of the M/L photopigment and are known as the “three-site rule”^47, 55^. For instance, a change at the site 180 from an Alanine for a Serine shift the absorbance peak in +7 nanometres; at the site 277, a phenylalanine for a Tyrosine shift +8 nanometres, and at the site 285 a change of an Alanine for a Threonine shift the peak of absorbance in +15 nanometres. In the case of an opposite substitution, it is possible to subtract the constant for the site and obtain an approximate sensitivity peak. The site 294 is also known to shift the predicted peak in spectral sensitivity in Atelids and was also verified^56^. Examining these sites it is possible to identify five major types of photopigments found in New World monkeys. Sequencing from both forward and reverse strands from, at least, two independent PCRs were used to determinate the type of M/L photopigment. Fragments were edited and mapped to the reference opsin gene (GenBank NM000513)^57^ analysed using Geneious^58^. Double peaks in the chromatogram were used to assign the individual as a homozygote, heterozygote, or hemizygote employing a specific plugin of Geneious software (Heterozygotes)^58^. Individuals assigned as double heterozygotes (i.e. double peaks at 180, 277, or 285) were not considered.
